# Oral Cancer Awareness of Tertiary Education Students and General Public in Singapore

**DOI:** 10.1016/j.identj.2022.11.021

**Published:** 2023-01-13

**Authors:** Pujan Rai, Charlene E. Goh, Francine Seah, Intekhab Islam, Wendy Wang Chia-Wei, Philip Martin Mcloughlin, John Ser Pheng Loh

**Affiliations:** a Discipline of Oral and Maxillofacial Surgery, Faculty of Dentistry, National University of Singapore, Singapore; b Discipline of Primary Dental Care & Population Health, Faculty of Dentistry, National University of Singapore, Singapore; c Discipline of Discipline of Endodontics, Operative Dentistry and Prosthodontics, Faculty of Dentistry, National University of Singapore, Singapore; d Department of Dental Medicine, Karolinska Institutet, Stockholm, Sweden

**Keywords:** Public health, Prevention, Cancer and precancer, Oral oncology, Risk factors, Dental students

## Abstract

**Objectives:**

Oral cancer confers high morbidity and mortality rates. Late diagnosis of oral cancer is linked to a lack of awareness of its existence and known risk factors. The purpose of this survey was to examine the knowledge and awareness of oral cancer amongst different groups in Singapore.

**Methods:**

A self-administered questionnaire (including questions on awareness, risk factor knowledge, and health beliefs about oral cancer) was distributed to undergraduate students from the medical and dental schools and other faculties at the National University of Singapore, as well as the general public.

**Results:**

A total of 470 responses were analysed. Both medical and dental students were almost universally aware of the disease and correctly identified recognised risk factors for oral cancers. Dental students had a significantly higher level of knowledge of chewing betel quid as a risk factor than medical students (98% vs 74%; *P* < .0001), although 1 in 10 dental students did not identify alcohol as a risk factor. In contrast, undergraduate students from other faculties were the least aware of oral cancer (62%). Within the general public, knowledge of the risk factors of oral cancer aside from smoking was low, with only 41% aware of viruses as a possible aetiology. However, the younger population group, aged 18 to 34 years old, in general had better knowledge of the risk factors of oral cancer compared with older participants.

**Conclusions:**

There is a general lack of awareness about oral cancer and its associated risk factors amongst certain cohorts of the Singapore population. There exists room for further targeted education.

## Introduction

Oral cancer is a common malignancy with high morbidity and mortality rates; however, general public awareness remains limited. Every year, 377,713 new cases are reported worldwide, representing 2% of all new cancer cases.^1^ There are, however, very wide regional variations, and Asia has the highest incidence of oral cancer of all continents,^2^ which may be closely linked to local lifestyles and cultural practices. Although the well-established aetiologic factors associated with oral cancer such as smoking, betel quid, and alcohol are avoidable,^3^, ^4^, ^5^ many individuals continue to engage in these behaviours.^6^ The incidence of oral cancer is relatively low in Singapore, with a 5-year prevalence of oral cancer in all age groups estimated at 12.79 per 100,000.^7^ However, this will likely change as immigration patterns, a major driver of Singapore's population growth, evolve with increased immigration from other countries including India, where oral cancer accounts for as much as 30% of all cancers.^8^ In addition, an emerging incidence of oral cancer associated with human papillomavirus (HPV) in the younger population has been observed worldwide, especially in developed countries, and is likely to increase the burden of oral cancer in Singapore in the coming years.^9^^,^^10^

Despite advancements in diagnosis and treatment of oral cancer, the overall 5-year survival rate of patients has been reported to be between 40% and 63%.^11^^,^^12^ Due to lack of awareness and delays in referral, the majority of oral cancer cases are diagnosed at an advanced stage,^13^ and in spite of experiencing debilitating surgery, radiotherapy, chemotherapy and financial burden, patients’ prognosis often remains limited. However, if detected early, oral cancer can often be treated successfully with low morbidity and good functional outcomes.^14^

Due to the significant morbidity and impact on quality of life, efforts to prevent the disease and increase early detection are necessary. Awareness of risk factors coupled with diagnostic tools to detect early presenting features can contribute to reducing the burden of oral cancer,^15^ and campaigns to increase oral cancer awareness and knowledge of the public have proven effective in detecting premalignant conditions and early-stage malignant disease.^16^ However, multiple studies to date have reported an alarming lack of knowledge about oral cancer and its risk factors, amongst both the general public and health care professionals.^17^^,^^18^ Likewise, some studies have found that undergraduate students in medical and dental schools have a general lack of training and knowledge about oral cancer.^19^, ^20^, ^21^

To the best of our knowledge, there is no prior study examining the knowledge and awareness of oral cancer in Singapore. Thus, the objective of this survey was to perform a baseline assessment of the awareness, knowledge, and health beliefs regarding oral cancer amongst several groups in Singapore. We sought to examine the awareness of medical and dental students, as they represent the next generation of clinicians with the greatest responsibility for early detection, and potential gaps in their education should be identified and addressed early. In addition, as the prevalence of oral cancer in the younger age groups increases globally and many of the aetiologic risky behaviours for oral cancer (eg, smoking, alcohol) are formed in young adulthood, we also sought to understand the awareness of general (ie, nonmedical/nondental) university students. Finally, we sought to assess the awareness of the general public in Singapore, including middle- to older-aged adults, so as to identify potential areas of intervention for future oral cancer awareness campaigns and targeted public health programmes.

## Methods

This survey was reviewed and approved by the National University of Singapore Institutional Review Board (NUS-IRB-2020-365). A cross-sectional survey was carried out between March 2020 and May 2021. We recruited participants from several groups as described above: medical, dental, general (nonmedical/nondental) university students at the National University of Singapore and the general public.

All participants were to be recruited in person for completion of the questionnaire. However, recruitment techniques had to be altered due to the COVID-19 pandemic. University students were mainly approached to participate in the survey via email blasts through different sports/social clubs and student groups. Following the easing of COVID-19 restrictions, students were approached in person at various locations within the campus, for example, canteens, outside libraries, and rest areas. Recruitment of the general public started only after COVID-19 restrictions were relaxed and was conducted in person only. Effort was made to recruit participants from various public locations including housing estates and shopping districts in order in order to better approximate the Singapore general public.

A commonly used questionnaire (Appendix A) that has been used in several studies conducted both internationally and in the Southeast Asian region^22^^,^^23^ was used to evaluate the oral cancer knowledge and awareness. We additionally included questions on the sociodemographic data of the participants including age, gender, ethnicity, housing type (commonly used as a proxy for socioeconomic status in Singapore), citizenship status, and how the participants preferred to receive information about oral cancer. The questionnaire was assessed for face validity by the research team before use and translated from English by professional translators to Mandarin, Bahasa Melayu, and Tamil, to represent the 4 official languages of Singapore. All questionnaires were administered electronically (Qualitrics XM) or provided as a hard-copy form upon request. The questionnaires were anonymously self-administered; however, the research assistant was available for assistance when required.

The sample size was calculated to estimate the prevalence of oral cancer awareness. The anticipated prevalence of oral cancer awareness was set at 50% with a 95% confidence interval and 10% precision level. The calculated sample size was 74 each for medical and dental undergraduates groups and 97 each for general undergraduates and general public groups. A finite population of 300 was considered for medical and dental undergraduates.

Descriptive statistics on oral cancer awareness, knowledge of risk factors, and health beliefs were presented by participant groups, and comparisons between the groups were made using the chi-square test or Fisher exact test with significance assessed at the *P* < .05 level. To gain further understanding of the sociodemographic factors associated with awareness, knowledge, and health beliefs, exploratory bivariate analysis was conducted only within the combined general population group consisting of the general (nonmedical/nondental) university students and general public participants.

## Results

### Participant demographics

A total of 549 participants completed the survey. After removing incomplete (n = 67), erroneous (n = 9 wrong population group selected), or consent-withdrawn (n = 3) surveys, a total of 470 complete responses from the university student groups (n = 95 dental, n = 61 medical, n = 165 general) and the general public (n = 149) were available for final statistical analysis. The mean age of the student respondents was 22 ± 2 years, and 44 ± 13 years for the general public respondents. The majority of participants were female (62%). Additional demographic data are presented in Table 1.

**Table 1:** Demographic characteristics of the participants (n = 470).

Characteristics	No. (%)
**Sex**	
Male	180 (38%)
Female	290 (62%)
**Ethnicity**	
Chinese	391 (83%)
Malay	28 (6%)
Indian	38 (8%)
Other*	13 (3%)
**Age, y**	
18–34	366 (78%)
35–54	68 (14%)
≥55	36 (8%)
**Citizenship**	
Singapore citizen	420 (89%)
Not a Singapore citizen	50 (11%)
**Housing type**†	
1–3 room public housing	44 (9%)
4–5 room and executive public housing‡	234 (50%)
Private housing	156 (33%)
Prefer not to say	28 (6%)
**Smoking status**	
Current smoker	15 (3%)
Former smoker	21 (4%)
Never smoker	434 (92%)
**Frequency of alcohol use**	
Several times a week or more	13 (3%)
Once a week or less	279 (59%)
Nondrinkers	178 (38%)
**Type of participants**	
Dental student	95 (20%)
Medical student	61 (13%)
Nonmedical/nondental student	165 (35%)
General public	149 (32%)

⁎ Includes Indonesian (eg, Batak), Burmese, Filipino, Japanese, Arab, Nepalese, Caucasian, and “prefer not to say.”

† n = 8 participants coded missing as responses including school dormitory, hostel, rentals.

‡ Includes 4–5 room Housing Development Board flat/Housing and Urban Development Corporation/executive flat (including maisonette)/executive condo/government landed; private housing includes private condominiums and landed housing.

### Oral cancer awareness

**Fig. 1:**
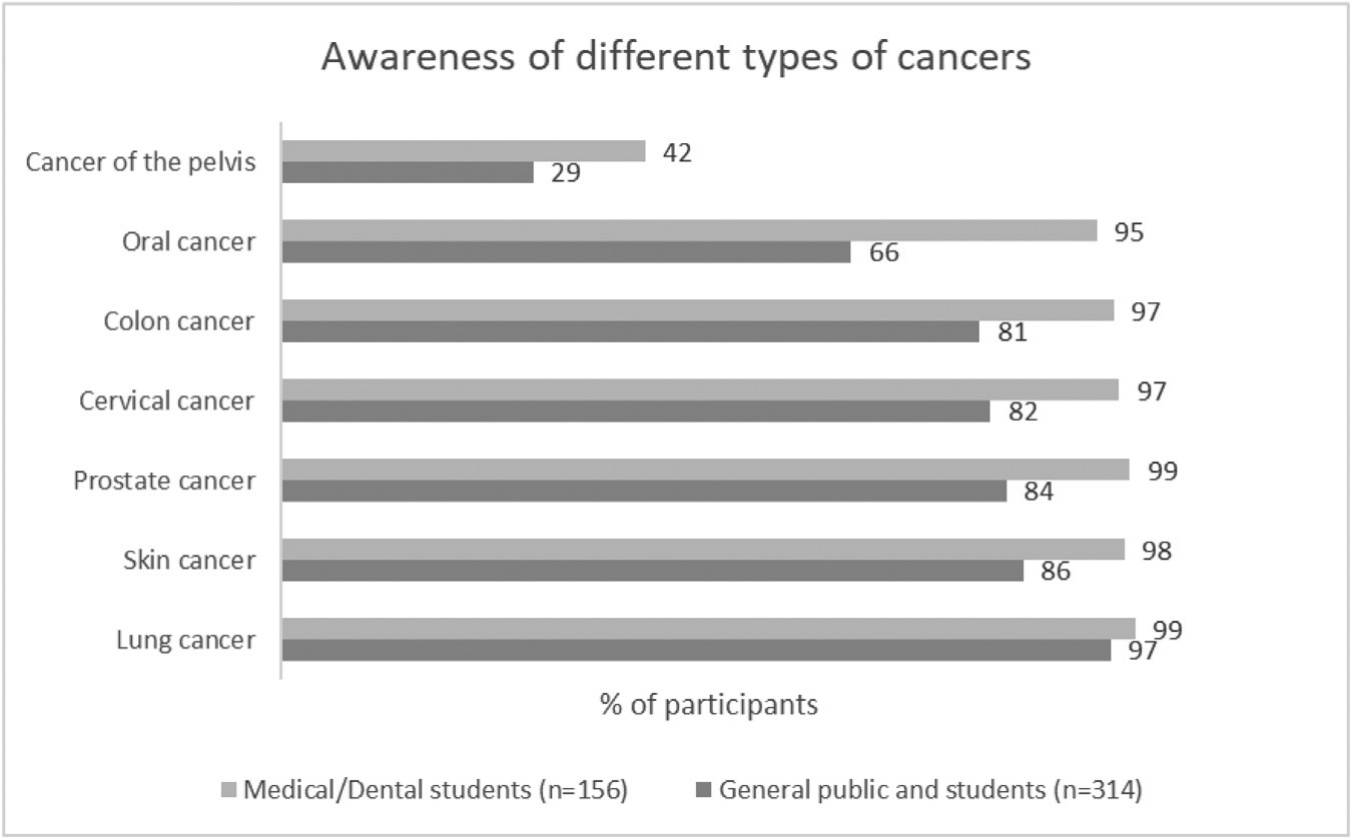
Awareness of different types of cancers as reported by participants.

When asked “Which of these types of cancer would you say you have heard of?”, only 66% of all participants from the combined general population were aware of oral cancer, and lung cancer was the most well-known type of cancer (Figure 1). Significant differences in oral cancer awareness were observed amongst groups (Table 2). Dental and medical students had similar awareness of oral cancer, 98% and 90% respectively, but significantly greater awareness compared to 71% of the general public and 62% of nonmedical/nondental students who were the least aware of oral cancer amongst the participant groups (*χ*^2^ test, *P*< .0001).

**Table 2:** Comparisons of oral cancer awareness, knowledge of risk factors, and health beliefs amongst the different groups of participants.

Type of participants	Dental students (n = 95)	Medical students (n = 61)	Dental vs medical students	Nonmedical/nondental student (n = 165)	General public (n = 149)	General students vs general public	Dental/medical vs nonmedical/nondental students	Dental/medical vs general public
			*P* value			*P* value	*P* value	*P* value
Q: Which of these types of cancer would you say you have heard of?								
Oral cancer awareness	98%	90%	.06	62%	71%	.08	<.0001	<.0001
Q:Thinking now just about oral cancer, here is a list of things which may or may not be linked with oral cancer. Select any of these which you think may be linked to oral cancer.								
Smoking	100%	97%	.15	92%	92%	.89	.003	.005
Car fumes	28%	43%	.05	40%	21%	.001	.22	.01
Alcohol use	84%	75%	.17	55%	42%	.02	<.0001	<.0001
Dental fillings	3%	18%	.002	18%	16%	.73	.02	.06
Chewing betel quid	98%	74%	<.0001	38%	56%	.001	<.0001	<.0001
Viruses	78%	82%	.54	50%	41%	.10	<.0001	<.0001
Q:Who develops cancer and who doesn't is a matter of chance, so there's nothing anybody can do to avoid it.								
Strongly disagree/disagree	81%	84%	.69	71%	52%	.001	.02	<.0001

*P* values from Pearson chi-square test or Fisher exact test.

### Knowledge of the risk factors of oral cancer

The majority of the general public participants (92%) identified smoking as a risk factor for oral cancer (Table 2). However, only 42%, 56%, and 41% of participants correctly recognised alcohol use, chewing betel quid, and viruses as risk factors, respectively. Some participants also incorrectly identified car fumes (21%) and dental fillings (16%) as risk factors for oral cancer.

Medical and dental students had better knowledge of all risk factors than all other groups (Table 2). Dental and medical students were overall similarly knowledgeable about oral cancer risk factors, though significantly more medical students wrongly identified dental fillings as a possible risk factor (*P* = .002), and dental students were significantly more aware of chewing betel quid as a risk factor (*P* < .0001; Table 2).

Knowledge of oral cancer aetiology was significantly different by age and ethnicity in exploratory analyses of the combined general population (Table 3). Indian ethnicity was associated with greater knowledge of chewing betel quid but less awareness of viruses as a risk factor (Table 3). Younger participants demonstrated significantly greater knowledge about the alcohol risk factors than those aged 35 to 54 years and 55 years and older (54%, 43%, 28%; *P*= .01), but less knowledge regarding chewing betel quid (42%, 59%, 53%; *P*= .04). Those aged 55 years and older had the least knowledge of oral cancer risk factors, and only 28% were aware of alcohol use as a risk factor (Table 3).

**Table 3:** Awareness of oral cancer, knowledge of risk factors, and health beliefs stratified by sociodemographic characteristics for participants from the general public and nonmedical/nondental students only (n = 314).

	Awareness of types of cancers		Risk factors*								Health beliefs†	
Demographics	Oral cancer		Smoking		Alcohol use		Chewing betel quid		Viruses		Chance	
	**%**	***P***	**%**	***P***	**%**	***P***	**%**	***P***	**%**	***P***	**%**	***P***
**Total**	**66%**	-	**92%**	-	**49%**	-	**47%**	**-**	**46%**	**-**		-
**Sex**		.18		**.02**		.05		.65		.18		.36
Male	71%		96%		42%		45%		41%		59%	
Female	63%		89%		53%		48%		49%		64%	
**Ethnicity**		.39		.25		.21		**.05**		**.01**		**.03**
Chinese	66%		92%		50%		47%		49%		65%	
Malay	58%		96%		58%		25%		54%		46%	
Indian	77%		93%		37%		63%		17%		60%	
Other†	54%		77%		31%		46%		46%		31%	
**Age, y**		.83		.77		**.01**		**.04**		.53		.07
18–34	66%		92%		54%		42%		48%		66%	
35–54	69%		91%		43%		59%		41%		51%	
≥55	64%		89%		28%		53%		42%		56%	
**Citizenship**		.34		.07		.11		.98		.39		.95
Singapore citizen	67%		93%		51%		47%		47%		62%	
Not a Singapore citizen	60%		84%		38%		47%		40%		62%	
**Housing type**‡		**.01**		.49		.71		.44		**.02**		.29
1–3 room HDB flat	72%		90%		46%		36%		36%		49%	
4–5 room HDB flat/executive flat and condo§	66%		93%		52%		49%		53%		61%	
Private housing (condominiums/landed)	72%		91%		45%		50%		34%		67%	
Prefer not to say	32%		84%		42%		42%		47%		63%	
**Smoking status**		.19		.32		.43		**.002**		.69		**.0003**
Current smoker	64%		100%		64%		0%		43%		14%	
Former smoker	47%		100%		42%		47%		37%		47%	
Never smoker	68%		91%		48%		49%		47%		65%	
**Frequency of alcohol use**		.87		.78		.16		.72		.76		.10
Several times a week or more	63%		88%		38%		63%		50%		38%	
Once a week or less	67%		93%		45%		46%		44%		66%	
Nondrinkers	65%		91%		55%		47%		48%		57%	

*P* values from Pearson chi-square test or Fisher Exact Test; **bold** text indicates <.05.

HDB, housing development board.

⁎ Response to the question, “Thinking now just about oral cancer, here is a list of things which may or may not be linked with oral cancer. Select any of these which you think may be linked to oral cancer.” Options given are smoking; car exhaust fumes; viruses; chewing betel quid (pan/paan); dental fillings; and alcohol..

† 100% and 99%, respectively, of participants responded strongly agree or agree to the statement, “Early detection of some cancers can improve the chances of successfully treating them” and “Some people can make changes in the way they live to reduce their risk of developing cancer” and are thus not shown. The percentages shown for those who responded strongly disagree or disagree to the statement, “Who develops cancer and who doesn't is a matter of chance, so there's nothing anybody can do to avoid it,” with a higher percentage reflecting positive health beliefs. ^†^Includes Indonesian (eg, Batak), Burmese, Filipino, Japanese, Arab, Nepalese, Caucasian, and “prefer not to say.”

‡ n = 8 participants coded missing as responses including school dormitory, hostel, and rentals.

§ Includes 4–5 room Housing Development Board flat/Housing and Urban Development Corporation/executive flat (including mansionette)/executive condo/government landed.

### Health beliefs about oral cancer

The health beliefs of the participants regarding oral cancer were overall positive. All but 2 of the 470 participants agreed with the statement that “Early detection of some cancers can improve the chances of successfully treating them.” Similarly, only 6 participants disagreed with the statement “Some people can make changes in the way they live to reduce their risk of developing cancer,” reflecting positive health beliefs.

On the other hand, the negative statement “Who develops cancer and who doesn’t is a matter of chance, so there’s nothing anybody can do to avoid it” had only 69% of all participants disagreeing. Whilst 81% and 84% of dental and medical students disagreed with this statement, nonmedical/nondental students, despite being of a similar age group, had a significantly lower percentage of disagreeing with this statement (71%; *P*= .02; Table 2). Participants from the general public had the lowest percentage, with only 52% disagreeing.

### *Preferred mode of receiving information on oral cancer*

**Fig. 2:**
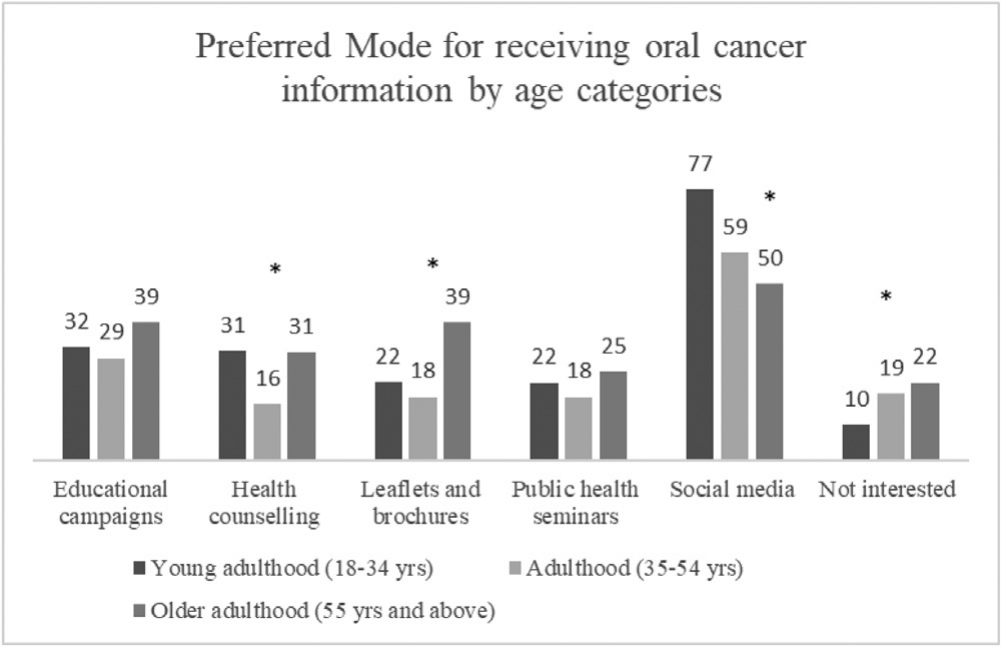
Preferred mode of receiving information about oral cancer by age groups in the general public and nonmedical/nondental students (n = 314). *P < .05 for chi-square test between age categories.

Figure 2 shows the modality for receiving oral cancer information preferred by the general public and nonmedical/nondental student respondents by age. Social media was the most popular method of obtaining information for all participants regardless of age, but particularly for those 18 to 34 years old, with 77% choosing this method, compared to 59% in adulthood and 50% in older adulthood. General health education campaigns or leaflets were tied for the second most preferred methods for older adults, whereas those aged 18 to 34 years preferred both educational campaigns and health counselling by health care workers during a consultation.

## Discussion

The present survey demonstrates that public awareness of oral cancer is limited when compared to other common cancers. Whilst the majority of participants in the general public have heard of oral cancer (66%), it is the second least known cancer in our survey. Breast cancer and lung cancer remain the best-known malignancies worldwide and can be considered as benchmarks for public awareness.^22^^,^^24^ In Singapore, our survey found that although the 5-year prevalence of skin cancer is only one-quarter that of oral cancer,^7^ 86% of the general public and nonmedical/nondental university students respondents were aware of skin cancer compared to only 66% aware of oral cancer. Whilst similar to other estimates in the region—with only 53% of adults in Jakarta, Indonesia, having heard of oral cancer^23^—the awareness in our surveyed Singapore population is lower than the 74% and 84% oral cancer awareness previously reported in Australia and Malaysia, respectively.^25^^,^^26^ Thus, this survey identifies substantial room for creating greater awareness of oral cancer in Singapore.

Community education and early exposure to knowledge are decisive factors in raising awareness of oral cancer in the general public. One of the most commonly used health behaviour models, the Health Belief Model, has been applied in oral cancer awareness-raising programmes^27^^,^^28^ and forms a theoretical basis for how education and increased awareness can improve oral cancer prevention behaviours. For example, an awareness of the risk factors of oral cancer can increase the perceived susceptibility of an individual with any of these factors, who will then be motivated to self-detect early symptoms or visit a health care professional. Fortunately, similar to other studies from the region,^23^^,^^26^ a high percentage of participants from the general population (92%) associated smoking use as a risk factor for oral cancer in this survey. On the other hand, only half or less recognised alcohol, chewing betel quid, and viruses as possible risk factors for oral cancer. Whilst younger participants were generally more aware of the risk factors, many were not familiar with the association between HPV and oral cancer.^10^ This is alarming, as the incidence of HPV-related oral and oropharyngeal cancers cancer in younger populations without exposure to tobacco and alcohol is increasing, with some countries even considering vaccine programmes.^29^ Lower levels of knowledge with increasing age have been consistently observed in studies from Hong Kong,^30^ Germany,^31^ and the US,^32^ even though most non–HPV-related cancers are diagnosed in those aged 55 and older.^33^ Likewise in our survey, age older than 55 years was consistent with the least knowledge of risk factors for oral cancer, highlighting the need for greater education in these older age groups.

The incidence of oral cancer is likely to rise in Singapore with increased migration from high-prevalence countries such as China and India.^12^^,^^34^ Various studies have observed that migrant communities from South and Southeast Asia, including India, Pakistan, and Bangladesh, continue to adhere to the traditional and cultural practices of betel nut usage after migration, with the habit persisting even in the second generation.^6^ A recent study amongst Indian immigrants in Australia found that although participants had all used betel nut at least once, they had limited knowledge of its oral cancer risk.^35^ Further research is needed amongst these potential communities at risk in Singapore to understand the prevalence of such oral cancer risk practices and the facilitators and barriers in modifying such habits, as has been conducted in other countries.^36^

Early detection of oral cancer depends on adequate training and awareness of the risk factors of oral cancer by health care professionals. Several studies have shown higher awareness of oral cancer risk factors in dental students compared to medical students.^17^^,^^19^^,^^37^ For example, a recent study conducted in Malaysia found a significantly higher awareness of oral cancer in dental students (99% vs 86%).^37^ In our survey, both medical and dental students showed awareness of oral cancer and its risk factors at the highest level. However, we identified several potential gaps in their knowledge. Whilst smoking and alcohol are considered well-established major risk factors for oral cancer, and all dental students recognised the risk of tobacco smoking, 1 in 10 dental students did not consider alcohol consumption as a leading risk factor for oral cancer. This is consistent with other studies in which fewer dental students recognised the negative effects of alcohol consumption.^37^, ^38^, ^39^, ^40^, ^41^ For example, alcohol was considered a risk factor by 35% to 62% of Malaysian dental students^19^^,^^42^ and only 21% of Nepalese dental students.^42^ The previously inconsistent evidence base for alcohol use as an isolated factor in the aetiology of mouth cancer has strengthened in recent years, especially when in conjunction with smoking.^43^ Our finding may simply reflect the slow transition of the alcohol risk factor message into undergraduate teaching and highlights the need for reinforcing the dental curriculum. We also observed that medical students had significantly lower knowledge of betel nut chewing as a risk factor than dental students (74% vs 98%), consistent with previous studies.^37^ As primary care physicians are often the first and only point of contact with the health system for patients, identification of individuals at high risk for oral cancer will be important for early screening and referral and can be enhanced in the medical curriculum.

Aside from identifying several key health education messages for improved awareness and knowledge, our findings identified avenues for an effective oral cancer awareness campaign.^45^ Social media was the preferred mode of oral cancer knowledge for all groups, even for those of the oldest age group aged 55 years and older. This is contrary to the common perception that older adults prefer traditional sources of health information such as educational leaflets. A recent study in Singapore supports our results, finding that older adults appreciate the health information obtained through social media and informal sources, with some even valuing this more than information obtained from health care professionals and institutions.^44^ Another popular method for receiving health information across ages was through general health education campaigns. The integration of oral cancer awareness together with other health messages is recommended by the 2015 FDI policy statement on oral cancer that “national health policies should be developed for oral cancer prevention strategies through integration with wider health literacy and messages in interdisciplinary education programs.”^45^ Likewise, the World Health Organisation concept of “health-promoting universities”^46^ could be used to address the low awareness and knowledge observed in university students from nonmedical/nondental faculties. Suggestions provided by our respondents for other modes of information such as using videos and testimonies, documentaries, and a dedicated website for oral cancer information also point the way to creating an effective multicomponent oral cancer awareness programme.^47^

There are some limitations of this study. Our survey employed convenience sampling, and the representativeness and generalisability of our findings to the wider general Singapore population may be limited, especially for groups who were underrepresented (eg, Malay and Indians, nonresidents, non–university-attending young adults). Our exploratory analyses of the risk factors associated with awareness and knowledge in a general population may also be biased. Nevertheless, our survey respondents are likely more “health conscious” than the average person, resulting in an overestimation of oral cancer awareness. Despite these limitations, we believe that our pilot study provides an important baseline assessment of the oral cancer awareness and knowledge of several groups in Singapore and has meaningful findings for dental education and public health.

## Conclusions

A relative lack of knowledge and awareness of oral cancer amongst the Singapore public and general university students has been identified and can be used to guide future interventions to increase public awareness. Several gaps in the dental and medical curricula that can be strengthened have also been highlighted. Increased awareness of this disease and its causes, course, and outcomes will empower a community to seek earlier professional advice, receive a timelier diagnosis, and thus ensure better treatment outcomes.

Pujan Rai and Charlene E. Goh contributed equally to this article. Philip Martin Mcloughlin and John Loh Ser Pheng contributed equally to the formulation, supervision, and production of the research and eventual outputs.

## Conflict of interest

None disclosed.

## Appendix Supplementary materials

Image, application 1

## References

[1] Sung H, Ferlay J, Siegel RL (2021). Global Cancer Statistics 2020: GLOBOCAN estimates of incidence and mortality worldwide for 36 cancers in 185 countries. CA Cancer J Clin.

[2] Shield KD, Ferlay J, Jemal A (2017). The global incidence of lip, oral cavity, and pharyngeal cancers by subsite in 2012. CA Cancer J Clin.

[3] Warnakulasuriya S, Trivedy C, Peters TJ. (2002). Areca nut use: an independent risk factor for oral cancer: The health problem is under-recognised. BMJ.

[4] IARC Working Group on the Evaluation of Carcinogenic Risks to Humans (2010). Alcohol consumption and ethyl carbamate. IARC Monogr Eval Carcinog Risks Hum.

[5] Wyss A, Hashibe M, Chuang SC (2013). Cigarette, cigar, and pipe smoking and the risk of head and neck cancers: pooled analysis in the International Head and Neck Cancer Epidemiology Consortium. Am J Epidemiol.

[6] Petti S, Warnakulasuriya S. (2018). Betel quid chewing among adult male immigrants from the Indian subcontinent to Italy. Oral Diseases.

[7] International Agency for Research on Cancer. Singapore fact Ssheet. World Health Organization; 2020.

[8] Coelho KR. (2012). Challenges of the oral cancer burden in India. J Cancer Epidemiol.

[9] Warnakulasuriya S. (2009). Causes of oral cancer–an appraisal of controversies. Br Dent J.

[10] Yete S, D'Souza W, Saranath D (2018). High-risk human papillomavirus in oral cancer: clinical implications. Oncology.

[11] Montero PH, Patel SG. (2015). Cancer of the oral cavity. Surg Oncol Clin N Am.

[12] Warnakulasuriya S. (2009). Global epidemiology of oral and oropharyngeal cancer. Oral Oncol.

[13] Abati S, Bramati C, Bondi S, Lissoni A, Trimarchi M. (2020). Oral cancer and precancer: a narrative review on the relevance of early diagnosis. Int J Environ Res Public Health.

[14] Ford P, Farah C. (2013). Early detection and diagnosis of oral cancer: strategies for improvement. J Cancer Policy.

[15] Nagao T, Warnakulasuriya S. (2020). Screening for oral cancer: Future prospects, research and policy development for Asia. Oral Oncol.

[16] Lim K, Moles DR, Downer MC, Speight PM. (2003). Opportunistic screening for oral cancer and precancer in general dental practice: results of a demonstration study. Br Dent J.

[17] Carter LM, Ogden GR. (2007). Oral cancer awareness of undergraduate medical and dental students. BMC Med Educ.

[18] McLeod N, Saeed N, Ali E (2005). Oral cancer: delays in referral and diagnosis persist. Br Dent J.

[19] Awan KH, Khang TW, Yee TK, Zain RB. (2014). Assessing oral cancer knowledge and awareness among Malaysian dental and medical students. J Cancer Res Ther.

[20] Cannick GF, Horowitz AM, Drury TF, Reed SG, Day TA. (2005). Assessing oral cancer knowledge among dental students in South Carolina. J Am Dent Assoc.

[21] Keat RM, Makwana M, HE Powell, Poveda A, Albuquerque R. (2019). Assessing confidence in the understanding and management of oral cancer among medical and dental undergraduates at a UK university. Br Dent J.

[22] Warnakulasuriya KAAS, Harris CK, Scarrott DM (1999). An alarming lack of public awareness towards oral cancer. Br Dent J.

[23] Wimardhani YS, Warnakulasuriya S, Subita GP, Soegyanto AI, Pradono SA, Patoni N. (2019). Public awareness of oral cancer among adults in Jakarta. Indonesia. J Investig Clin Dent.

[24] Monteiro LS, Salazar F, Pacheco J, Warnakulasuriya S. (2012). Oral cancer awareness and knowledge in the city of Valongo. Portugal. Int J Dent..

[25] Zachar JJ, Huang B, Yates E. (2020). Awareness and knowledge of oral cancer amongst adult dental patients attending regional university clinics in New South Wales, Australia: a questionnaire-based study. Int Dent J.

[26] Ghani WMN, Doss JG, Jamaluddin M, Kamaruzaman D, Zain RB. (2013). Oral cancer awareness and its determinants among a selected Malaysian population. Asian Pac J Cancer Prev.

[27] Schliemann D, Donnelly M, Dahlui M (2018). The ‘Be Cancer Alert Campaign’: protocol to evaluate a mass media campaign to raise awareness about breast and colorectal cancer in Malaysia. BMC Cancer.

[28] Jeihooni AK, Dindarloo SF, Harsini PA. (2019). Effectiveness of health belief model on oral cancer prevention in smoker men. J Cancer Educ.

[29] Timbang MR, Sim MW, Bewley AF, Farwell DG, Mantravadi A, Moore MG. (2019). HPV-related oropharyngeal cancer: a review on burden of the disease and opportunities for prevention and early detection. Hum Vaccin Immunother.

[30] Adeoye J, Chu CS, Choi S-W, Thomson P. (2022). Oral cancer awareness and individuals’ inclination to its screening and risk prediction in Hong Kong. J Cancer Educ.

[31] Hertrampf K, Wenz H-J, Koller M, Wiltfang J. (2012). Public awareness about prevention and early detection of oral cancer: a population-based study in Northern Germany. J Craniomaxillofac Surg.

[32] Patton LL, Agans R, Elter JR, Southerland JH, Strauss RP, Kalsbeek WD. (2004). Oral cancer knowledge and examination experiences among North Carolina adults. J Public Health Dent.

[33] García-Martín JM, Varela-Centelles P, González M, Seoane-Romero JM, Seoane J, García-Pola MJ., Panta P (2019). Oral cancer detection: novel strategies and clinical impact.

[34] Yang Y, Zhou M, Zeng X, Wang C. (2021). The burden of oral cancer in China, 1990–2017: an analysis for the Global Burden of Disease, Injuries, and Risk Factors Study 2017. BMC Oral Health.

[35] Saraswat N, Prabhu N, Pillay R, Everett B, George A. (2022). Oral cancer risk behaviours of Indian immigrants in Australia: a qualitative study. Aust N Z J Public Health.

[36] Lokhande S, Glover M, Selket K. (2013). Chewing tobacco use among South-East Asian men in Auckland. Int J Migr Health Soc Care.

[37] Gunjal S, Pateel DGS, Lim RZS, Yong LL, Wong HZ. (2020). Assessing oral cancer awareness among dental and medical students of a Malaysian private university. Int Dent J.

[38] Frola MI, Barrios R. (2017). Knowledge and attitudes about oral cancer among dental students after Bologna plan implementation. J Cancer Educ.

[39] Hassona Y, Scully C, Abu Tarboush N (2017). Oral cancer knowledge and diagnostic ability among dental students. J Cancer Educ.

[40] Keser G, Pekiner FN. (2019). Assessing oral cancer awareness among dental students. J Cancer Educ.

[41] Kujan O, Alzoghaibi I, Azzeghaiby S (2014). Knowledge and attitudes of Saudi dental undergraduates on oral cancer. J Cancer Educ.

[42] Poudel P, Srii R, Marla V. (2020). Oral cancer awareness among undergraduate dental students and dental surgeons: a descriptive cross-sectional study. JNMA J Nepal Med Assoc.

[43] Ogden GR. (2018). Alcohol and mouth cancer. Br Dent J.

[44] Han M, Tan XY, Lee R, Lee JK, Mahendran R. (2021). Impact of social media on health-related outcomes among older adults in Singapore: qualitative study. JMIR Aging.

[45] FDI policy statement on oral cancer: adopted by the FDI General Assembly (2016). 24 September 2015, Bangkok, Thailand. Int Dent J..

[46] World Health Organization. The Edmonton Charter for Health Promoting Universities and Institutions of Higher Education. Edmonton: World Health Organisation; 2005.http://www.gesundheitsfoerdernde-hochschulen.de/Inhalte/E_Gefoe_HS_internat/2005_Edmonton_Charter_HPU.pdf. (Accessed 3 January 2023).

[47] Macpherson LMD. (2018). Raising awareness of oral cancer from a public and health professional perspective. Br Dent J.

