# Supplementary material for: Oral Cancer Awareness of Tertiary Education Students and General Public in Singapore
Source: Int Dent J. 2023 Jan 13;73(5):651–8. doi: 10.1016/j.identj.2022.11.021 (PMC10509411; doi:10.1016/j.identj.2022.11.021)
Supplement: Supplementary file 1 [file mmc1.docx]

**Online Supplemental Material**

**Appendix 1: Questionnaire**

Q1 Which of these types of cancer would you say you have heard of?
 *(please select all that applies)*

- Lung cancer (1)
- Skin cancer (2)
- Cervical cancer (3)
- Prostate cancer (4)
- Cancer of the colon (5)
- Oral cancer (6)
- Cancer of the pelvis (7)
- ⊗None of the above (8) [you should not have selected any other options]

Q2 Thinking now just about oral cancer, here is a list of things which may or may not be linked with oral cancer. Select any of these which you think **may be linked** to oral cancer: 
*(please select all that applies)*

- Smoking (1)
- Car exhaust fumes (2)
- Viruses (3)
- Chewing betel quid (Pan/ Paan) (4)
- Dental fillings (5)
- Alcohol (6)

Q3 Which of the following groups of people are **most likely** to have a greater chance of developing oral cancer? *(please select all that applies)*

- Heavy smokers (1)
- People who drink (alcohol) heavily (2)
- Men in their 50s (3)
- People 24-35 years old (4)
- People with a low income (5)
- People with a high income (6)
- Anyone over 70 years old (7)

Q4 To what extent do you think these statements about cancer are right or wrong?

|  | Strongly agree (1) | Agree (2) | Disagree (3) | Strongly disagree (4) |
| --- | --- | --- | --- | --- |
| i. Early detection of some cancers can improve the chances of successfully treating them |  |  |  |  |
| ii. Who develops cancer and who doesn't is a matter of chance, so there's nothing anybody can do to avoid it |  |  |  |  |
| iii. Some people can make changes in the way they live to reduce their risk of developing cancer |  |  |  |  |

**Participant information:**

Q5 Age

________________________________________________________________

Q6 Sex

- Male (1)
- Female (2)

Q7 Ethnicity

- Chinese (1)
- Malay (2)
- Indian (3)
- Others (please specify) (4) ________________________________________________

Q8 Citizenship status

- Singapore citizen (SC) (1)
- Permanent Resident of Singapore (SPR) (2)
- Neither SC or SPR, but based in Singapore (3)

Q9 Marital status

- Single (1)
- Married (2)
- Divorced (3)
- Separated (4)
- Widow (5)
- Prefer not to say (6)

Q10 What type of house do you live in?

- 1-2 room HDB flat (1)
- 3 room HDB flat (2)
- 4-5 room HDB flat/ HUDC/ Executive Flat (including Mansionette) (3)
- Private Residence (4)
- Others (please specify) (5) ________________________________________________
- Prefer not to say (6)

Q11 Which population group do you best represent?

- Dental student (1)
- Medical student (2)
- Non-dental/medical student (3)
- General public (4)
- NUH staff (medical) (5)
- NUH staff (non-medical) (6)

Q12a Smoking History

- Current Smoker (1)
- Past Smoker (2) [skip to Q13]
- Non-smoker (3) [skip to Q13]

Q12b On average, how many sticks a day do you smoke? (current smoker)

________________________________________________________________

Q13 Alcohol Use

|  | Almost every day (1) | Several times a week (2) | Once a week (3) | Less frequent (4) | None (5) |
| --- | --- | --- | --- | --- | --- |
| How often do you consume alcohol? |  |  |  |  |  |

Q14 Chewing of betel quid

|  | Almost every day (1) | Several times a week (2) | Once a week (3) | Less frequent (4) | None (5) |
| --- | --- | --- | --- | --- | --- |
| How often do you chew betel quid (Pan/ Paan)? |  |  |  |  |  |

Q15 Chewing tobacco

|  | Almost every day (1) | Several times a week (2) | Once a week (3) | Less frequent (4) | None (5) |
| --- | --- | --- | --- | --- | --- |
| How often do you chew tobacco? |  |  |  |  |  |

Q16 Which platform would you prefer to receive more information about oral cancer?

*(You can select more than one option)*

- General health education campaigns (e.g. roadshows) (1)
- Social media (e.g. Facebook, Instagram feeds) (2)
- Public health seminars or forums by relevant experts (3)
- Health education by healthcare worker during a consultation (4)
- Leaflets and brochures (5)
- Others (please specify) (6) __________________________________________
- ⊗I am not interested (7) [you should not have selected any other options]
